# Comparison of Remimazolam and Midazolam for Sedation During Endoscopic Retrograde Cholangiopancreatography‐Related Procedures

**DOI:** 10.1002/deo2.70281

**Published:** 2026-01-13

**Authors:** Yuki Tanisaka, Shomei Ryozawa, Suguru Ito, Masafumi Mizuide, Akashi Fujita, Ryuichi Watanabe, Ryosuke Hamamura, Yoshiki Matsuno

**Affiliations:** ^1^ Department of Gastroenterology Saitama Medical University International Medical Center Saitama Japan

**Keywords:** benzodiazepines, ERCP, midazolam, remimazolam, sedation

## Abstract

**Objectives:**

Remimazolam is an ultra‐short‐acting benzodiazepine recently approved for endoscopic procedures in Japan as a sedative agent. We aimed to compare the efficacy and safety of remimazolam and midazolam for endoscopic retrograde cholangiopancreatography (ERCP)‐related procedures.

**Methods:**

ERCP‐related procedures performed under sedation with remimazolam (between August 2025 and November 2025) and midazolam (between April 2025 and July 2025) were retrospectively reviewed. The primary outcome was the time from the end of the procedure to patient awakening and readiness for discharge. The secondary outcomes included sedation success throughout the procedure, time from the initial dose to achieving sedation, dosage, rate of antagonist use for awakening, and sedation‐related adverse events.

**Results:**

Eighty‐eight patients underwent ERCP‐related procedures with remimazolam and 86 with midazolam. The median times from the end of the procedure to patient awakening and readiness for discharge with remimazolam and midazolam were 2 min (interquartile range [IQR], 1–3) and 4 min (IQR, 3–5), respectively. Sedation with remimazolam required a shorter time to awaken than sedation with midazolam (*p* < 0.01). Furthermore, the rate of antagonist use for awakening was significantly lower with remimazolam than with midazolam (*p* < 0.01). The success rate of sedation, median time from the initial dose to achieving sedation, and rate of sedation‐related adverse events were not significantly different between groups (*p* = 0.49, 0.13, and 0.27, respectively).

**Conclusions:**

Remimazolam demonstrated significantly shorter time to patient awakening, suggesting a safer and more efficient discharge process after ERCP‐related procedures.

**Abbreviations**
BMI: body mass index
ERCP: endoscopic retrograde cholangiopancreatography
IQR: nterquartile range

## 1 Introduction

Endoscopic retrograde cholangiopancreatography (ERCP)‐related procedures are essential for both the diagnosis and treatment of pancreatobiliary disorders [1, 2]. Furthermore, owing to the complexity of these procedures, including balloon enteroscopy‐assisted ERCP, which has emerged as an effective technique for patients with surgically altered anatomy, prolonged procedure times are often required, making effective and safe sedation essential [3, 4, 5]. Benzodiazepine agonists are commonly used as sedative agents for endoscopic procedures. Among them, midazolam has been widely used as a sedative agent during endoscopic procedures [6]. However, it has a relatively long half‐life [7], and its sedative effects may persist even after the procedure is completed. This prolongs the time to discharge and increases the need for extended observation and additional human resources. Although flumazenil, as a benzodiazepine antagonist, can be used to help patients awaken after prolonged sedation, resedation due to the lingering effects of midazolam remains a significant concern [8]. Furthermore, there are potential adverse reactions to flumazenil, such as sweating, flushing, and nausea/vomiting [9].

Remimazolam (Anerem; Mundipharma K.K., Tokyo, Japan) is an ultra‐short‐acting benzodiazepine agonist that exerts sedative effects via gamma‐aminobutyric acid type A receptors [10]. It was initially approved in Japan only for the induction and maintenance of general anesthesia. It has now also been approved for use in endoscopic procedures. Several reports have demonstrated the efficacy and safety of remimazolam for endoscopic procedures, including in ERCP‐related procedures [11, 12, 13, 14]. All of these studies reported that remimazolam was safe and well‐tolerated during endoscopic procedures, with no serious adverse events observed, regardless of the type of procedure performed.

As midazolam has long been used as the standard sedative for endoscopic procedures, it is important to compare the efficacy and safety of remimazolam with those of midazolam. Although several studies have compared remimazolam and midazolam for upper gastrointestinal endoscopy and colonoscopy in Japanese patients [15, 16], no studies have specifically evaluated this comparison for ERCP‐related procedures. ERCP‐related procedures are often complex and require prolonged sedation, making such a comparison essential to determine whether remimazolam can be used as a first‐line sedative. Therefore, we conducted a study to compare the efficacy and safety of remimazolam with those of midazolam during ERCP‐related procedures.

## 2 Methods

### 2.1 Study Design

This retrospective study was conducted at a tertiary referral care center in Japan that performs more than 600 ERCP‐related procedures annually. This study was approved by the Institutional Review Board of Saitama Medical University International Medical Center (number: 2025‐130). All patients provided written informed consent for the ERCP‐related procedures and sedation. The need for informed consent was waived because the patients could use the opt‐out method on the Institutional Review Board website. This study was conducted in accordance with the principles of the Declaration of Helsinki.

### 2.2 Patients

The key inclusion criteria for this study were as follows: (1) patients who underwent ERCP‐related procedures under sedation between April 2025 and November 2025; (2) patients with a body mass index (BMI) of <30 kg/m^2^. The key exclusion criteria were as follows: (1) regular use of or tolerance to benzodiazepines; (2) history of paradoxical reactions or disinhibition to benzodiazepine agonists that required switching to propofol during previous ERCP‐related procedures.

### 2.3 Sedative Procedure During ERCP‐Related Procedures

Patients were placed in the prone position before sedation. Vital signs, including blood pressure, pulse rate, SpO_2_, respiratory rate, and electrocardiography results, were continuously monitored. Capnography was also used to detect episodes of apnea. Procedures were performed using the JF‐260 V or TJF‐Q290 (Olympus Marketing, Tokyo, Japan). Balloon enteroscopy‐assisted ERCP was performed using SIF‐H290 (Olympus Marketing, Tokyo, Japan) with a working length of 152 cm and a working channel of 3.2 mm diameter for patients with surgically altered anatomy, including Roux‐en‐Y gastrectomy, hepaticojejunostomy with Roux‐en‐Y, pancreaticoduodenectomy, and Billroth II gastrectomy.

Remimazolam became available at our institution at the beginning of August 2025 and was used for all ERCP‐related procedures from August to November 2025. Midazolam was used exclusively between April and July 2025. No crossover of sedative use occurred during the study period.

Immediately after the administration of 35 mg pethidine hydrochloride, remimazolam or midazolam was administered intravenously as a single initial dose. For remimazolam, a single intravenous dose of 3 mg was administered over 15 s as the initial dose. A single intravenous dose of 2 or 3 mg midazolam was administered at the discretion of the endoscopist. For elderly patients aged ≥75 years or those who were underweight (<45 kg), the remimazolam dose could be reduced by half at the physician's discretion, whereas the midazolam dose could be reduced by 1 mg. The level of sedation was assessed 2 min after administration using the Modified Observer's Assessment of Alertness/Sedation (MOAA/S) scale (Table 1) [17]. If sedation (MOAA/S score ≤3) was appropriately achieved, the endoscope was inserted. If adequate sedation was not achieved (MOAA/S score ≥4), an additional 1 mg of remimazolam or midazolam was administered, and the sedation level was reassessed 2 min later. This process was repeated until an MOAA/S score ≤3 Was achieved. For remimazolam, the number of additional doses was limited to a maximum of five, as recommended by the pharmaceutical company.

**TABLE 1:** Modified Observer's Alertness/Sedation (MOAA/S) scale [17].

MOAA/S scale	Description
5	Responds readily to a name spoken in a normal tone
4	Responds lethargically to a name spoken in a normal tone
3	Responds only after the name is called loudly and/or repeatedly
2	Responds only after mild prodding or shaking
1	Responds only after a painful trapezius squeeze
0	No response after a painful trapezius squeeze

After the endoscopic procedure began, if the patient showed signs of arousal (MOAA/S score of 5 or body movements) and the physician judged additional sedation necessary, an extra dose of 1 mg remimazolam or 1–2 mg midazolam was administered, with at least a 2‐min interval from the preceding dose.

If adequate sedation was not achieved despite additional dosing before endoscope insertion, or if paradoxical reactions or disinhibition occurred requiring a switch to propofol during the procedure, the study drug was deemed ineffective and failed.

After completion of the procedure, the patient's level of awakening was assessed every minute using the Aldrete score [18] to determine the readiness for discharge (Table 2). An Aldrete score of 9 or higher was considered indicative of adequate awakening and readiness for discharge. Post‐procedure assessments were performed by Dr. Akashi Fujita and Dr. Ryuichi Watanabe, both of whom are experienced in ERCP‐related procedures and procedural sedation. These assessors were involved throughout both study periods. They were not blinded to the sedative used. Flumazenil, a benzodiazepine antagonist, was administered to patients with delayed recovery at the physician's discretion. As all patients were hospitalized, they were monitored by nursing staff in their beds for at least two hours.

**TABLE 2:** Aldrete scores [18].

Outcome measures	Description	Score
Respiration	Able to take deep breaths; cough reflex present	2
	Respiratory distress or shallow breathing noted	1
	Apnea present	0
SpO_2_	SpO_2_ > 92% (room air)	2
	SpO_2_ 92%, or more (with supplemental oxygen)	1
	SpO_2_ < 90% (with supplemental oxygen)	0
Consciousness	Awakening	2
	Awakens to verbal stimulation	1
	Unresponsive to voice/pain	0
Circulation	Blood pressure within ±20 mmHg from the pre‐procedure baseline	2
	Blood pressure ±20–50 mmHg from the pre‐procedure baseline	1
	Blood pressure over ±50 mmHg from the pre‐procedure baseline	0
Activity	Able to move the limbs	2
	Able to move any two limbs	1
	Unable to move any limbs	0

### 2.4 Definitions and Outcome Measurement

We compared the patient characteristics and sedation‐related outcomes of ERCP‐related procedures performed under sedation with remimazolam versus midazolam. Since most previous studies evaluating sedation‐related outcomes with remimazolam have reported its advantage of rapid recovery [12, 13, 14, 15, 16], we defined the primary outcome of this study as the time from the end of the procedure to patient awakening and readiness for discharge. The secondary outcomes included sedation success throughout the procedure, number of doses until initiation of the procedure, time from the initial dose to achieving sedation, number of doses required throughout the procedure, dosage of each sedative, procedure time, number of doses required throughout the procedure, dosage of pentazocine, rate of flumazenil use for awakening, and sedation‐related adverse events.

Sedation success throughout the procedure was defined as achieving and maintaining adequate sedation, both at the initiation of the procedure and throughout the procedure, without any of the aforementioned issues. Procedure time was defined as the time from scope insertion to removal. Sedation‐related adverse events were defined as any unfavorable events that occurred in patients who received remimazolam or midazolam.

### 2.5 Statistical Analysis

Categorical variables were evaluated as numbers and percentages, and differences were evaluated using Fisher's exact probability test. Continuous variables were evaluated as median and interquartile range (IQR), and differences were evaluated using the Mann‐Whitney U‐test. Statistical significance was set at *p* < 0.05. All statistical analyses were performed using EZR version 1.61 (Saitama Medical Center, Jichi Medical University, Saitama, Japan) [19].

## 3 Results

### 3.1 Patients

**FIGURE 1:**
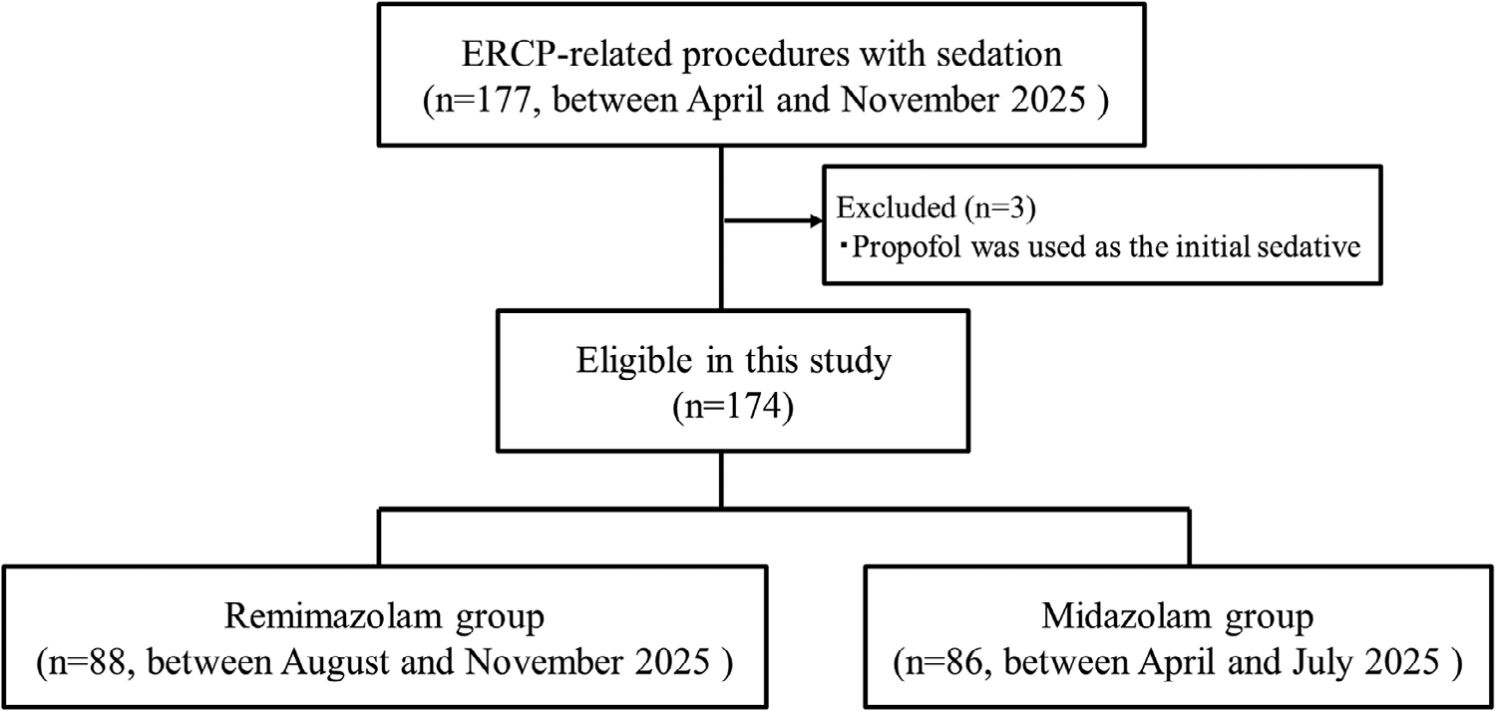
Flow diagram of the study. *n*, number.

Of the 177 patients who underwent ERCP‐related procedures under sedation, three were excluded (one from the remimazolam group and two from the midazolam group) because propofol was used as the initial sedative. In total, 174 patients were included in this study (Figure 1). Among them, 88 patients underwent ERCP‐related procedures under remimazolam sedation, and 86 patients underwent ERCP‐related procedures under midazolam sedation. Table 3 shows the characteristics of patients who underwent ERCP‐related procedures under sedation. There were no significant between‐group differences in any variables. A balloon enteroscopy‐assisted procedure was performed in over 20% of patients in both groups.

**TABLE 3:** Characteristics of patients who underwent endoscopic retrograde cholangiopancreatography (ERCP)‐related procedures.

	Remimazolam group (*n* = 88)	Midazolam group (*n* = 86)	*p*‐value
Sex (male), *n* (%)	66 (76.7)	55 (64.0)	0.14
Age, median (IQR), years	77 (68.8–80)	76 (67–81)	0.68
Height, median (IQR), cm	163 (156–167)	163 (155.3–167)	0.88
Weight, median (IQR), kg	54.6 (46.5–63.6)	53.8 (49.4–60)	0.89
BMI, median (IQR), kg/m^2^	20.6 (19.1–22.7)	21.1 (19.3–23.5)	0.19
ASA category, *n* (%)			0.41
I	45 (51.1)	54 (63)	0.13
II	38 (43.2)	30 (34.9)	0.28
III	5 (5.7)	2 (2.3)	0.44
IV–VI	0	0	1
Presence of alcohol intake (yes), *n* (%)	27 (30.7)	26 (30.2)	0.75
Presence of smoking (yes), *n* (%)	35 (39.8)	30 (34.9)	0.53
Indication, *n* (%)			0.62
Biliary drainage^#^	60 (68.2)	56 (65.1)	0.35
Stone extraction in the bile duct^#^	24 (27.3)	30 (34.9)	0.35
Cholangioscopy‐guided procedure	8 (9.1)	4 (4.7)	0.95
Balloon enteroscopy‐assisted procedure	19 (21.6)	24 (27.9)	0.38

Abbreviations: ASA, American Society of Anesthesiologists; BMI, body mass index; IQR, interquartile range; *n*, number.

^#^ Including cholangioscopy‐guided and balloon enteroscopy‐assisted procedures.

### 3.2 Sedation‐Related Outcomes of the ERCP‐Related Procedures

Table 4 summarizes the sedation‐related outcomes of the ERCP‐related procedures. For the primary outcome, the median time from the end of the procedure to patient awakening and readiness for discharge was 2 min (IQR, 1–3 min) in the remimazolam group and 4 min (IQR, 3–5 min) in the midazolam group. Sedation with remimazolam resulted in a significantly shorter awakening time than that with midazolam (*p* < 0.01). Furthermore, the rate of flumazenil use for awakening was significantly lower with remimazolam than with midazolam (*p* < 0.01). The median time from the initial dose to sedation was 3 min (IQR, 2–4 min) in the remimazolam group and 2 min (IQR, 2–3 min) in the midazolam group. There was no significant difference between the groups (*p* = 0.13).

**TABLE 4:** Sedation‐related outcomes of the endoscopic retrograde cholangiopancreatography (ERCP)‐related procedures.

	Remimazolam group (*n* = 88)	Midazolam group (*n* = 86)	*p*‐value
Sedation success throughout the procedure, *n* (%)	88 (100)	85 (98.8)	0.49
Median pre‐procedure dose, mg (IQR)	3 (3–3)	2 (2–3)	
Median number of doses until the initiation of the procedure, times (IQR)	1 (1–2)	1 (1–1)	0.21
Median time from the initial dose to achieving sedation, min (IQR)	3 (2–4)	2 (2–3)	0.13
Median number of doses required throughout the procedure, times (IQR)	3 (2–5)	2 (1–3)	0.01
Median time from the end of the procedure to patient awakening and readiness for discharge, min (IQR)	2 (1–3)	4 (3–5)	<0.01
Median total dose, mg (IQR)	5 (4–7)	3 (3–4)	
Median procedure time, min (IQR)	29 (20–46.8)	30.5 (20.3–45.8)	0.8
Use of flumazenil for delayed awakening after the procedure, *n* (%)	4 (4.5)	27 (31.4)	<0.01
Median dose of pethidine, mg (IQR)	35 (35–35)	35 (35–35)	0.86
Sedation‐related adverse events, *n* (%)	2 (2.3)	5 (5.8)	0.27
Hypotension	1 (1.1)	1 (1.1)	1
Heart rate reduction to below 40 bpm	1 (1.1)	1 (1.1)	1
Hypoxemia (SpO_2_ <90%)	0	3 (3.5)	0

Abbreviations: IQR, interquartile range; *n*, number.

The sedation success rates in the remimazolam and midazolam groups were 100% and 98.8%, respectively, with no significant difference between the two groups (*p* = 0.49). One patient in the midazolam group developed disinhibition after the procedure began, requiring a switch to propofol to continue the procedure. This case was defined as an unsuccessful sedation. The median number of doses until the initiation of the procedure was 1 time (IQR, 1–2 times) in the remimazolam group and 1 time (IQR, 1–1 time) in the midazolam group, with no significant difference between the groups (*p* = 0.21). The median number of doses required throughout the procedure was 3 times (IQR, 2–5 times) in the remimazolam group and 2 times (IQR, 1–3 times) in the midazolam group, with a significant difference between the groups (*p* = 0.01). The median dose of pethidine was not significantly different between groups (*p* = 0.86). Median procedure time was 29 min (IQR, 20–46.8 min) in the remimazolam group and 30.5 min (IQR, 20.3–45.8 min) in the midazolam group, with no significant difference between the groups (*p* = 0.80).

Sedation‐related adverse event rates were 2.3% in the remimazolam group (one case of hypotension, and one case of bradycardia with a heart rate <40 bpm) and 5.8% in the midazolam group (one case of hypotension, one case of bradycardia with a heart rate <40 bpm, and three cases of hypoxemia [SpO_2_ < 90%]), with no significant difference between the groups (*p* = 0.27). Fortunately, all sedation‐related adverse events observed in this study were mild in severity. Cases of hypotension and bradycardia resolved completely after rapid intravenous fluid administration, whereas cases of hypoxemia resolved after increasing oxygen from 2 to 4 L/min (Supporting Information).

## 4 Discussion

To the best of our knowledge, this is the first study to compare remimazolam and midazolam for sedation during ERCP‐related procedures. Sedation‐related outcomes were evaluated to determine whether remimazolam can be used as a first‐line sedative. There were no significant between‐group differences in any of the patient characteristics, providing a favorable basis for comparison.

This study demonstrated that remimazolam resulted in a significantly shorter time from the end of the procedure to patient awakening and readiness for discharge compared with midazolam. This suggests that its clinical benefits are attributable to its pharmacokinetic profile as an ultra‐short‐acting agent. Previous studies have also reported that remimazolam leads to a rapid recovery after endoscopic procedures [12, 13, 14, 15, 16]. Two Japanese studies compared sedation‐related outcomes between remimazolam and midazolam in upper gastrointestinal endoscopy and colonoscopy [15, 16]. Both studies demonstrated faster patient recovery after endoscopic procedures, consistent with our findings. This advantage may help reduce the need for prolonged post‐procedure observation and lessen the burden on the staff responsible for monitoring patients after the procedure. Furthermore, the rate of flumazenil use for awakening was significantly lower in the remimazolam group than in the midazolam group. Because flumazenil can cause adverse reactions such as sweating, flushing, nausea, vomiting, hiccups, agitation, and seizures [9], it is preferable to minimize its use whenever possible. Therefore, based on our findings of shorter awakening times and the reduced need for flumazenil, remimazolam may contribute to a safer and more efficient discharge process after ERCP‐related procedures.

Most other outcomes, including sedation success throughout the procedure, number of doses until initiation of the procedure, procedure time, and pentazocine dosage, showed no significant differences between the two groups. Furthermore, the median time from the initial dose to sedation achievement did not differ significantly between the remimazolam and midazolam groups. Midazolam has long been used as a standard sedative in endoscopic procedures and has demonstrated reliable efficacy. In our study, remimazolam did not show inferior sedative effectiveness. By contrast, the median number of doses required throughout the procedure was significantly higher in the remimazolam group than in the midazolam group. This finding also reflects the pharmacokinetic profile of remimazolam as an ultra‐short‐acting agent.

There were no significant differences in sedation‐related adverse events between groups. Remimazolam was not inferior in terms of safety. Notably, no cases of hypoxemia (SpO_2_ < 90%) occurred in the remimazolam group, whereas three cases were observed in the midazolam group, although this difference was not statistically significant. A previous report evaluating sedation‐related outcomes of remimazolam for ERCP‐related procedures also presented no cases of hypoxemia [14]. Furthermore, a recent study reported that the remimazolam group required less supplemental oxygen during upper gastrointestinal endoscopic procedures than the midazolam group [16]. These findings, coupled with ours, suggest an advantage attributable to the shorter duration of action of remimazolam.

This study has several limitations. First, this was a retrospective study, which could not eliminate selection bias. Second, there were a few patients with an American Society of Anesthesiologists classification of ≥3 in either group, indicating that further evaluation of remimazolam is needed in patients with more severe comorbidities or poorer overall health. Furthermore, because the assessors who performed the post‐procedure evaluations were not blinded, assessment bias may have influenced the primary outcome. Therefore, further studies involving patients with a wider range of clinical conditions undergoing ERCP‐related procedures and blinded assessment for post‐procedure evaluations are warranted.

## 5 Conclusion

Remimazolam demonstrated significantly shorter times to patient awakening, with no loss of sedative efficacy and no increase in sedation‐related adverse events, suggesting a safer and more efficient discharge process after ERCP‐related procedures compared with that of midazolam. Although further studies are warranted, remimazolam may be considered as a potential first‐line sedative for ERCP‐related procedures.

## Author Contributions

The paper was authored by Yuki Tanisaka, who designed and drafted the article. Shomei Ryozawa, Suguru Ito, Masafumi Mizuide, Akashi Fujita, Ryuichi Watanabe, Ryosuke Hamamura, and Yoshiki Matsuno provided critical revision of the article for important intellectual content. Akashi Fujita interpreted the statistical analysis. Yuki Tanisaka finally approved the article for submission. The final version of the manuscript was approved by all authors.

## Conflicts of Interest

The authors declare no conflicts of interest.

## Funding

None to report.

## Ethics Statement

**Approval of the research protocol by an Institutional Reviewer Board**: This research was approved by the Institutional Review Board at Saitama Medical University International Medical Center (number: 18‐278).

## Consent

Written informed consent was obtained from all patients prior to undergoing ERCP. Patients were given the opportunity to opt out of participation through a notice posted on the hospital's website.

## Clinical Trial Registration

None to report.

## Supporting information




**Supporting File 1**: deo270281‐sup‐0001‐SuppMat.pdf

**Supporting File 2**: deo270281‐sup‐0002‐SuppMat.pdf

**Supporting File 3**: deo270281‐sup‐0003‐SuppMat.pdf

**Supporting File 4**: deo270281‐sup‐0004‐SuppMat.pdf

**Supporting File 5**: deo270281‐sup‐0005‐SuppMat.pdf

**Supporting File 6**: deo270281‐sup‐0006‐SuppMat.pdf

**Supporting File 7**: deo270281‐sup‐0007‐SuppMat.pdf

**Supporting File 8**: deo270281‐sup‐0008‐SuppMat.pdf
